# Optimizing targeting strategies for lithotripsy through in-vitro and in vivo studies with consideration of respiratory regularity

**DOI:** 10.1186/s12894-024-01422-x

**Published:** 2024-03-21

**Authors:** Sung Yong Cho, Hyeji Park, Jae Suk Park, Seong Chan Kim, Oh bin Kwon, Hyun jae Song, Min Joo Choi

**Affiliations:** 1 Department of Urology, Seoul National University Hospital, Seoul, Korea; 2 Seoul National University College of Medicine, Seoul, Korea; 3 Interdisciplinary Postgraduate Program in Biomedical Engineering, Jeju National University, Jeju, Korea; 4 Department of Electronic Engineering, Sogang University, Seoul, Korea; 5 Department of Medicine, School of Medicine, Jeju National University, Jeju, Korea

**Keywords:** Respiration, Respiratory regularity, Extracorporeal shockwave therapy, Lithotripsy, Treatment outcome

## Abstract

**Background:**

This work aimed to identify a method to achieve improved stone targeting and safety in shockwave lithotripsy by accounting for respiration.

**Methods:**

We set up an electromotive device simulating renal movement during respiration to place artificial stones within the phantom gel, measuring stone weight changes before and after shockwave exposure and the cavitation damage. We conducted clinical trials using respiratory masks and sensors to monitor and analyze patient respiration during shockwave lithotripsy.

**Results:**

The in vitro efficiency of lithotripsy was higher when adjusted for respiration than when respiration was not adjusted for. Slow respiration showed the best efficiency with higher hit rates when not adjusted for respiration. Cavitation damage was also lowest during slow respiration. The clinical study included 52 patients. Respiratory regularity was maintained above 90% in regular respiration. When respiration was regular, the lithotripsy rate was about 65.6%, which stayed at about 40% when respiration was irregular. During the lithotripsy, the participants experienced various events, such as sleep, taking off their masks, talking, movement, coughing, pain, nervousness, and hyperventilation. The generation of shockwaves based on respiratory regularity could reduce pain in patients.

**Conclusion:**

These results suggest a more accurate lithotripsy should be performed according to respiratory regularity.

## Introduction

Extracorporeal shockwave lithotripsy (ESWL) has become the preferred treatment for urinary stone removal during the last 40 years. ESWL is still gaining popularity due to its minimal invasiveness of requiring local anesthesia and shorter treatment duration than the more invasive endoscopic or open surgery alternatives. However, the literature suggests that stone-free rates of ESWL range from 32 to 90% and 43–90%, respectively [1, 2]. Residual stone fragments after unsuccessful ESWL can lead to several complications, including urinary infection, obstruction, and hematuria. Renal hematoma can also occur in severe cases, although it is rare.

Determining the optimal ESWL settings is desirable to improve its success rates and minimize the associated health risks. Meta-analyses and systematic reviews of randomized controlled ESWL trials have suggested that the optimal frequency of ESWL to improve stone fragmentation efficiency is 60 to 90 times per minute [3]. However, even with an optimal frequency, it is intuitive that the kidney and the stone will move with breathing. As a result, the shockwave accuracy suffers, and kidney and perirenal tissues are thus exposed to shockwave-derived damage caused by respiratory motion. Further, reducing the accuracy of stone targeting due to positional change by breathing can lead to inefficient lithotripsy, causing side effects [4]. Therefore, ESWL can be applied more effectively by considering the breathing pattern of patients, thus allowing shockwaves to be more accurately transmitted to stones.

The efficiency and safety of ESWL with kidney stone movement have yet to be thoroughly investigated. While it is intuitive that respiration can compromise the accuracy of ESWL targeting, there remains uncertainty regarding the efficacy and safety of lithotripsy when adjusting for respiration compared to when not adjusted. This study offers evidence for the clinical application of an introduced method that precisely targets shockwaves by considering respiratory patterns and identifying specific respiratory points to enhance the efficiency and safety of lithotripsy. This approach introduces a novel method for improving the accuracy of shockwave targeting by considering respiration patterns, thereby providing a foundation for its clinical implementation. This study modulated a practical ESWL approach to achieve better stone targeting during lithotripsy through in-vitro experiments and a clinical investigation accounting for respiratory motion. We suggest a novel approach that contributes to improved outcomes of ESWL therapy of stones by considering the respiratory regularity of the patients.

## Materials and methods

### Preparation of artificial stones, phantom gel, and a respiration-electromotive device

**Fig. 1:**
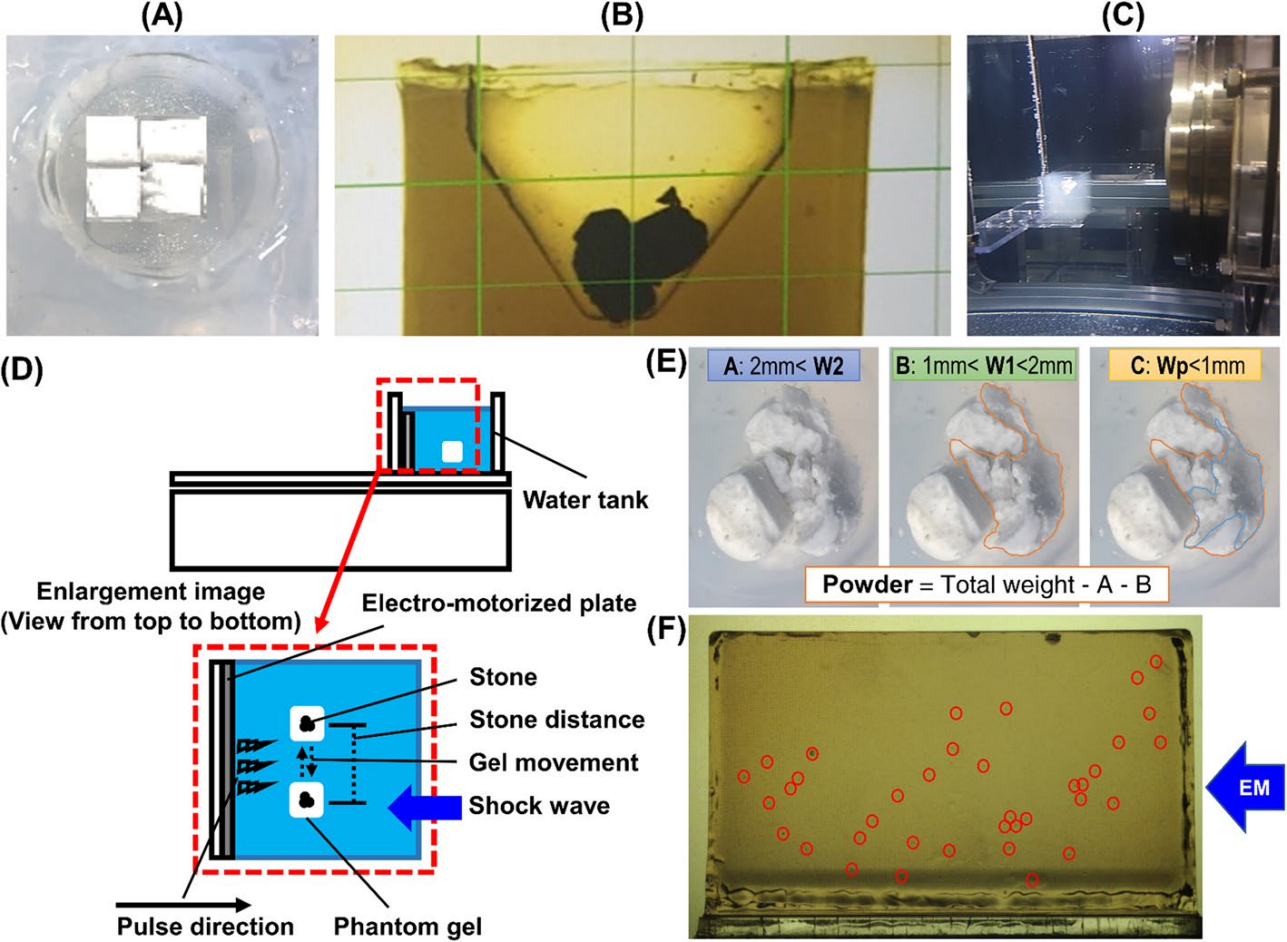
**a** Top view of the four artificial stones in the phantom gel. **b** Outside view of the phantom gel with artificial stones. **c** Respiration-electromotive device setup showing the phantom gel on the motorized plate submerged in water. **d** A respiration-electromotive device consisting of a motorized positioner in a water tank. **e** Stone decomposition divided into powder, 1 mm, and 2 mm by size. W0 (initial weight) is soaked in water and weighed after removing all excess water. Before weighing, fragmented stones were filtered with 2- and 1-mm sieves and dried to remove excess water. W1, W2, and Wp labeled according to size, where the particles > 2 mm are large particles W2, particles 1–2 mm are small particle W1, and fragmented stone powder is Wp. Stone fragment marked as W0; initial, W2; > 2 mm, W1; 1–2 mm, and Wp; W2 subtracted W1. **f** Image contrast was adjusted using commercial graphic software to mark and measure cavitation on the gel, enhancing visualization

By analyzing the responses of kidney stones using phantom gel, which mimics the surrounding kidney tissue [5], we investigated how respiration affects the efficiency of lithotripsy and the damage to the gel. To reproduce urinary calculi in their natural environment, we prepared artificial stones and phantom gel, which represent normal kidneys responsive to pressure as described by Lafon et al. and Guntur and Choi [6, 7]. Four artificial stones were placed in 4 × 4 × 4 mm phantom gels (Fig. 1A) with a mean hardness of 91.2 ± 15.8 N/m2 as measured by a push-pull gauge to resemble the mechanical properties of calcium oxalate monohydrate stones [8]. The gel with an inner side space for stone placement was positioned on a motorized plate (Fig. 1B). A respiration-electromotive device consisting of a motorized positioner in a water tank was set up to simulate kidney movement during respiration (Fig. 1C). It was custom-designed to fit the gel with low walls on three sides and hold it in place so that it would be unmoved at the force of shockwave delivery, as shown below in a simple illustration (Fig. 1D).

The options were set according to human respiratory rates (RR) at three (20 RR/min), four (15 RR/min), or five seconds per cycle (12 RR/min) for fast, intermediate, and slow breathing, respectively. For lithotripsy, shockwave frequency sets of 60 times/min (1 Hz), 90 times/min (1.5 Hz), and 120 times/min (2 Hz) are most commonly used in clinical practice. There are stone fragmentation condition sets for two situations. In one situation, the shockwaves and the position of the stone were temporally matched, and the shockwaves only hit the stone when it was at its starting place of translational movement. In the other situation, shockwaves were fired at a constant frequency of 60, 90, or 120 Hz regardless of the stone position. For each experiment, the stones were treated with 150 shockwaves at 14 KeV, an intermediate energy level that is commonly used in clinical practice. It duplicated respiration and frequency in 27 trials of 3 sets of artificial stones multiplied by three kinds of respiration and frequency. Each condition was repeated and carried out back-to-back.

### Measurement of stone fragment efficiency and gel cavitation damage on the phan-tom gel

Four artificial stones were soaked in water and weighed after removing all excess water (W0). After delivering the shock, the stones were removed and collected from the phantom gel by carefully spraying water. Next, the fragments were filtered sequentially through 2- and 1-mm sieves and allowed to air dry for five minutes to eliminate excess water. The particles > 2 mm and the ones sized 1 to 2 mm were labeled W2(A) and W1(B), respectively (Fig. 1E). Fragmented stone powder (Wp, C) was assumed to have been lost during shock delivery; therefore, it was calculated by subtracting W2 and W1 from W0. A JAB1004 analytical balance (Joan Lab, Zhejiang, China) capable of detecting a range from 10 mg to 120 g was used for stone weight measurement. Finally, the authors macroscopically counted the focused shockwaves firing on the stone (Fig. 1F), and the fraction of the total number of shocks was represented as the hit rate.

The phantom gel was imaged using an EOS 5D Mark III camera (Canon, Tokyo, Japan) from a lateral perspective after shock delivery. Commercial graphic software was used to mark and measure cavitation on the gel after adjusting image contrast for better visualization.

### Clinical setting

In total, 52 patients who received ESWL participated. This study was approved by the institutional review boards of our institution (IRB: 2003-046-1108). Among the patients who underwent ESWL, cases in which the stones were located in the upper urinary tract and had diameters ranging from 4 to 15 mm were included for analysis. However, patients with urinary tract obstruction, hemorrhagic diseases that are difficult to treat with drugs and pregnant or pediatric patients were excluded from the study [4].

**Fig. 2:**
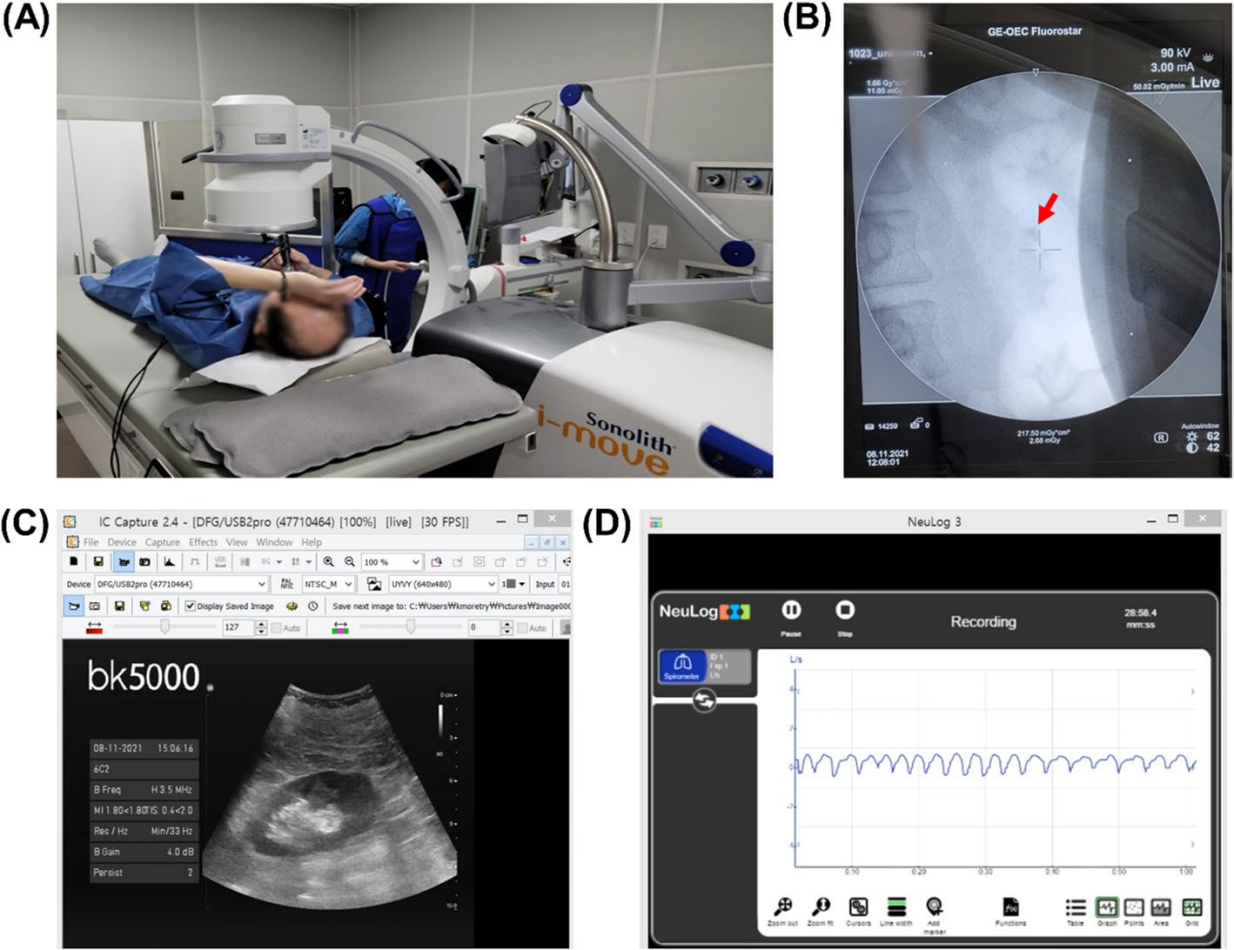
Evaluation of respiration. **a**, **b** The position of the bed was set to fit the shockwave using X-rays, the operator explained to the patient the reason for measuring respiration during lithotripsy, then lithotripsy began. **c**, **d** Patients’ respiration curves of inhalation and exhalation were recorded, and the locations of the stones in the kidney were predicted using ultrasound to recognize a specific breath point and generate high-accuracy shockwave targeting

Informed consent was obtained from all patients. After guiding the patient to the lithotripsy bed (Fig. 2A), the investigators adjusted the position of the bed to fit the shockwave (Fig. 2B), explained to the patient the reason for measuring respiration during lithotripsy, and then started lithotripsy. If the patient felt discomfort, they might take a break, or the investigators might adjust the shockwave power. However, ESWL was stopped early if the patient complained of severe pain. About 2 or 3 weeks after lithotripsy, a non-contrast kidney CT scan was taken to confirm whether the calculus was shattered. During the ESWL process, the complications and the degree of pain were checked repeatedly to verify safety.

### Evaluation of respiration

Patients’ respiration curves were recorded to predict stone locations using ultrasound. This helped identify a specific breath point for accurate shockwave targeting, enhancing lithotripsy efficiency. Corrections were made during the ESWL process for events that might disrupt respiration.

ESWL was performed using a Sonolith i-move® shockwave lithographer (EDAP, Lyon, France), and high-definition Bk5000 Type2300® Ultrasound (BK Medical, Peabody, Massachusetts) was used (Fig. 2C). A breath measurement sensor (Spirometer logger sensor NUL_216 with USB Module USB-200) (Neulog) and an ultrasound device were connected to a research laptop, and a video recording program (o-Cam) was used to analyze movement distance of stones during the ESWL (Fig. 2D). To collect respiratory data, a respiratory mask (large: MA501, small: MA502) (Mow medical, Wonju, Republic of Korea) and a respiratory measurement sensor were connected to the patient’s oral cavity. The facial connection between the respiratory mask and the respiratory measurement sensor was checked and sealed with tape to minimize air leakage.

The respiration measurement sensor was checked and zeroed before data measurement. During the 30-minute ESWL procedure, the patient’s breathing was continuously recorded. Respiration was measured every 0.1 s in L/sec. Ultrasound and X-ray images were captured for 5 to 10 s to simultaneously measure breathing-induced movement and the maximum longitudinal diameter of the stones.

### Parameters

Respiratory regularity was defined as 90% or more of regular breathing among all respirations. Therefore, even if cough, sleep, mask off, tension, hyperventilation, conversation, and body movement occurred, it was defined as respiratory regularity if it was maintained at over 90%. Respiratory regularity was determined by dividing the respiration measurement time minus the event occurrence time by the respiration measurement time.

$$\ast Respiratory\;regularity\;(\%)\;=\;(Respiration\;measurement\;time\;(sec)\;-\;Event\;occurence\;time\;(sec))\;/\;Respiration\;measurement\;time\;(sec)\;\times\;100$$

After the procedure, the stone fragmentation rates were compared between regular and irregular breathing patients.

$$\ast Stone\;fragmentation\;efficiency\;(\%)\:=\:Fragments\;or\;Stationary\;/\;(fragments\;+\;stationary)\;\times\;100$$

The pain degrees were classified as none, mild, middle, or severe. The number of patients demonstrating each pain classification was observed.

### Statistical analysis

Descriptive statistics were conducted to analyze the characteristics of the patient group, and the Mann-Whitney U test was used to compare the two groups. The variables were analyzed to determine the associations between the relation to the actual value (cm) of the kidney travel distance, age, gender, the location of stones, laterality, and events during the process. Chi-square analysis was applied to verify the association between each category. IBM SPSS Statistics ver. 25.0 (IBM Co., Armonk, New York, USA) was used, and a two-tailed *p*-value < 0.05 was considered to indicate statistical significance. Univariate and multivariate logistic regression analyses were conducted, calculating odds ratios with 95% CIs to estimate the associations between respiration regularity and lithotripsy efficiency, adjusting for potential confounders.

## Results

### Stone fragmentation efficiency on the phantom gel

**Fig. 3:**
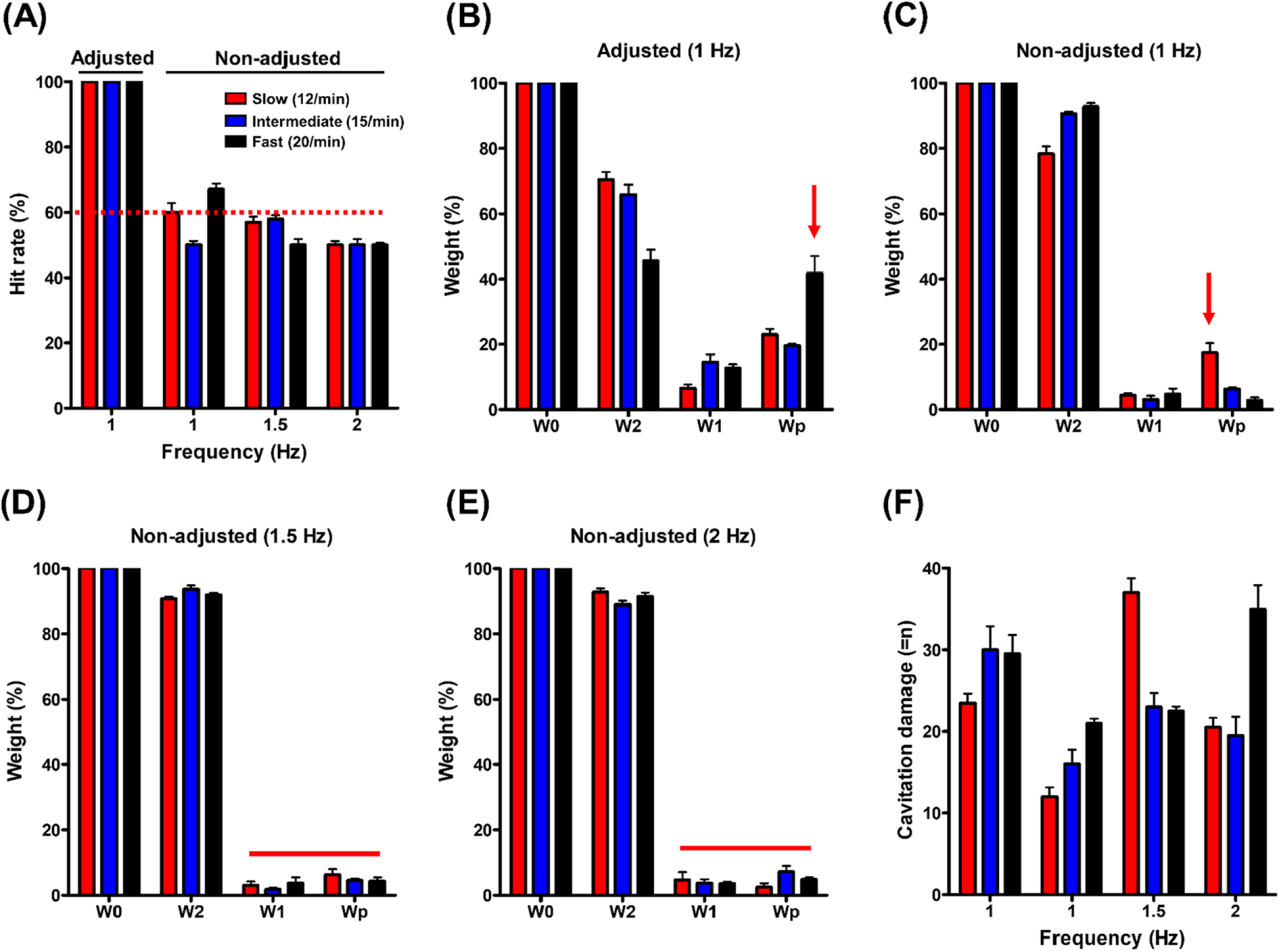
Analysis of stone fragment efficiency. **a** The hit rate quantification of stone targeting was measured by adjusted respiration compared to non-adjusted respiration. Shock-wave frequency: 60 times/min (1 Hz), 90 times/min (1.5 Hz), and 120 times/min (2 Hz). Respiration rate: slow (12 RR/min), intermediate (15 RR/min), and fast (20 RR/min). Hit rate: a number of shock-waves focused on the stone out of a total number of shock-waves. **b** The change in the weight of the stone was measured according to respiration rate and shock-wave frequency. The stone weight change was analyzed by adjusted respiration (**b**), compared to non-adjusted respiration with frequency of 1 Hz (**c**), 1.5 Hz (**d**), and 2 Hz (**e**). **f** A percentage of phantom gel cavitation numbers was counted according to respiration adjustment. Phantom gel cavitation was individually marked and counted using a Sprinter-HD high-speed camera of the CamRecord-Sprinter series (Optronics GMBH, Ludwigstr, Kehl, Germany

Figure 3A summarizes the results regarding the 100% hit rate of the respiration-adjusted lithotripsy. The hit rate is the number of shockwaves focused on the stone out of the total. Without respiration adjustment, the 1 and 1.5 Hz frequencies showed higher hit rates than those of 2 Hz. The hit rate ranged from 50 to 67% in the respiration-electromotive device setting.

Figure 3B (upper mid) summarizes the results of the respiration adjustment. The percentage of exchanged stone weight calculated the stones’ fragmentation efficiency. When adjusted for respiration and applying the shockwave frequency to 1 Hz, the stones’ fragmentation efficiency increased according to the increase in respiration cycles. The fractions of fragments W2, W1, and Wp were represented in the order of slow RR (70.4%, 6.6%, 23.0%), intermediate RR (65.9%, 14.6%, 19.5%), and fast RR (45.5%, 12.8%, 41.8%) respiration. The highest particles of Wp were fast respiration.

Without respiration adjustment in shockwave frequency applied in the order of 1 Hz (Fig. 3C, upper right), 1.5 Hz (Fig. 3D, under left), and 2 Hz (Fig. 3E, under mid), the stones’ fragmentation efficiency was found to be highest in slow respiration. The fraction of large fragments W2 measured in the slow (78.3%, 79.3%, and 90.7%), intermediate (90.7%, 93.6%, and 91.9%), and fast (92.7%, 88.9%, and 91.4%) respiration. The remaining small particles W1 for slow, intermediate, and fast RR were less than 5%. The opposite result was observed with the fraction of powder Wp, where the slow respiration showed the largest fraction of 7.9–17.3%, contrasting a range from 2.5 to 7.3% for the other two respiration conditions. Slow respiration showed the best fragmentation to Wp at all frequencies.

### Patient and overall trial performance

After receiving 58 patients, six were excluded from the experiments because of device errors. As a result, 52 patients (33 males and 19 females) were analyzed in the respiration data set. The mean age was 55 ± 16 years. The stone laterality was 29 patients on the left and 23 patients on the right side, and the mean kidney size was 10.0 ± 1.0 cm. The mean maximal diameter and number of stones were 9.9 ± 3.9 mm, and 1.2 ± 0.6 stones, respectively. The ESWL treatment was conducted for 32 and 14 patients in the first and second sessions, respectively. The mean number of transmitted shockwaves was 2,900 ± 281, and the mean of the total energy was 744.63 ± 105.54 KeV. The respiratory motion range was from 6 to 30 mm, with an average value of 12.8 ± 4.8 mm. The mean maximum volumes of inhalation and exhalation are 0.96 ± 0.4 L and − 1.23 ± 0.57 L, respectively. The mean ESWL execution time and the mean ESWL number were 28.6 ± 2.7 min, 1.65 ± 1.23 times. The mean respiration flow velocity and respiration rate(bpm) were 0.79 ± 0.3, 17.69 ± 0.4, and the mean respiratory duration was 3.4 ± 0.6.

### Lithotripsy efficiency according to respiratory regularity

Among the 52 patients, 33 (63.5%) were identified as having respiratory regularity, whereas 19 (36.5%) had respiratory irregularity. Respiratory regularity from 90 to 95% was shown in eight patients (24.2%), and respiratory regularity from 95 to 100% in 25 patients (75.8%). By contrast, irregularities from 80 to < 90% were shown in seven patients, 50 to < 80% in six patients, and < 59% in four patients. Among patients with respiratory regularity > 90%, two patients with non-specific breathing cycles of very slow respiration > 5 s and very rapid breathing shorter than 2 s were excluded from the regular breathing group.

The regular breathing group showed fragmented stones in 22 patients (66.7%) and stationary in 11 patients (33.3%), while the irregular group showed fragmented stones in seven (36.8%) and stationary in 12 patients (63.2%). The stone fragmentation efficiency was 29.9% higher in the regular group than in the irregular group (OR 3.43, 95% CI 1.05–11.16, *P* = 0.036*). Table 1 shows that the kidney migration (OR 12.14, 95% CI 1.04-141.71, *P* = 0.046*), the respiration regularity > 95% (OR 6.10, 95% CI 1.04–35.97 and *P* = 0.046*), and the intensity (OR 1509.06, 95% CI 1.51-1510472.04 and *P* = 0.038*) were significant predictors for stone-free status.

**Table 1:** Univariate and multivariate logistic regression analysis for prediction of stone-free status after shockwave lithotripsy

		Univariate			Multivariate	
Variables	OR	95% CI	*p* value	OR	95% CI	*p* value
Age	0.935	0.809–1.079	0.356			
Height	1.236	0.912–1.676	0.172			
Weight	0.822	0.608–1.111	0.202			
Laterality	0.047	0.001–1.941	0.107			
Max size	1.001	0.667–1.504	0.995			
ESWL time	4.413	0.229–85.162	0.326	1.656	0.967–2.835	0.066
ESWL No	0.586	0.031–10.971	0.721			
Total energy	1.019	0.973–1.068	0.428			
Intensity (Hz)	890246.377	0.000-6.026E + 18	0.363	1509.061	1.508-1510472.035	0.038*
Respiration number	0.414	0.134–1.275	0.124	0.853	0.613–1.187	0.346
Respiration regularity (> 95%)	401.806	0.336-480114.589	0.097	6.101	1.035–35.966	0.046*
Respiration event number	1.122	0.553–2.275	0.751			
Respiration duration	0.003	0.000-1.531	0.068			
Kidney migration	3715.476	0.799-17283214.50	0.056	12.137	1.039-141.705	0.046*
Kidney size	0.840	0.191–3.688	0.818			

* *p* < 0.05

### Respiratory irregularity and associated events

During the ESWL procedures, five types of events occurred: (1) cough, (2) sleep, (3) mask off, (4) tension or hyperventilation, and (5) conversation or body movements; some patients exhibited two or more events simultaneously. Table 2 summarizes the events in patients with regular and irregular breathing. In regular respiration, cough, hyperventilation, and conversation showed the highest number in cases of < 10%, while in irregular respiration, sleep and tension showed the highest number.

**Table 2:** Changes in respiration waveforms according to events and degree of event occurrence. Some patients showed two or more events

Event	Waveform	Note	Cases in the regular respiration group (N)	Cases in the irregular respiration (N)
**Cough**		**Sharp wavy appear down**	**5**	**1**
**Sleep**		**Respiratory waveform is shallow breathing cycle is constant**	**2**	**6**
**Mask off**		**The end of the breathing waveform become straight cow**	**3**	**4**
**Tension** **Hyperventilation**		**Respiratory waveform is high** **Irregular breathing cycle**	**6**	**8**
**Conversation** **Movement**		**Prolonged breathing cycle** **Respiratory waveform is high**	**11**	**2**
**Respiration Regularity (%) < 95 ~ 100**		**Respiration waveform is regular**		
**Respiration Regularity (%) 90 ~ 95**				

The group with > 95% of regular breathing exhibited a consistent waveform, and the group with < 95% showed a slight bounce from time to time. In this group, the duration of an event was very short, and the frequency was low. On the other hand, an irregular waveform appeared when an event occurred, such as a cough with a sharp wave, sleep with shallow breathing, conversation and movement with prolonged breathing, etc.

### Safety represented by phantom gel damage, complications and degree of pain

The cavitation damage in the respiration-adjusted group was lowest in slow respiration compared to intermediate and fast respirations (23.5 vs. 30 and 29.5, respectively). Without respiration adjustment, no definite differences existed among different respirations (Fig. 3F).

In the clinical investigation, no patients had a high fever, acute pyelonephritis, significant bleeding, or perirenal hematoma. The pain strengths of none, mild, moderate, and severe appeared in 4, 11, 18, and 0 cases in the regular group and 1, 4, 10, and 4 cases in the irregular group, respectively (*P* = 0.019*). However, the prescription rate of drugs on the procedure day showed no significant differences in 17 cases out of 33 patients (51.5%) in the regular group and 12 cases out of 19 patients (63.2%) in the irregular group (*P* = 0.563).

## Discussion

### Respiratory regularity and its effect on the stone fragmentation efficiency

Regardless of the change in respiratory movement, regularly-triggered shockwaves showed an inefficient lithotripsy process that reduced the number of shockwaves delivered to stones, which may cause tissue damage [4, 5, 9]. Some studies have considered the respiratory movement of the kidney to be the cause of unsuccessful lithotripsy and proposed some practical techniques [10]. There are tips and tricks to reduce the effect of kidney motion, such as the self-controlled breathing technique [8], the use of an abdominal compression plate [11], quantification of the range of kidney motion in conscious patients [12], and respiratory modulation in the anesthetized patients [13]. However, none of them are commonly used in the urology field. The stone being a moving target suggests that respiratory rate, excursion length, shockwave rate, and such may counteract the effort of stone fragmentation. To our knowledge, no studies have investigated the use of respiratory regularity and shockwave generation according to the stone position at the start of the shockwave treatment. The result of the current investigation would help increase the stone fragmentation efficiency. Respiratory regularity is more critical than kidney migration. Therefore, respiratory regularity increased the efficiency of lithotripsy by increasing the respiratory regularity more than having less migration. Consequently, the stone-free rate is higher in regular and high kidney migration than in irregular and low kidney migration.

The authors have attempted to improve stone fragmentation efficiency by using artificial stones, laser, or therapeutic ultrasound [8, 14, 15]. In this study, the hit rates of shockwaves to the stones on the phantom gel were only 50–67% of those in the adjusted respiration. These results are similar to a previous clinical investigation showing as much as 40% missed shockwaves in clinical practice [13]. According to the respiratory regularity in this study, the stone fragmentation of the regular respiration group was 29.9% higher than that in the irregular group. All the comparisons of the ESWL outcomes according to each respiratory duration and shockwave generation settings were impossible. However, more than 63.5% of patients presented regular breathing during ESWL, and 76% showed very high regularity (> 95%). By including the two patients with very slow and very fast respirations in the regular group, the portion of respiratory regularity could be increased to 67.3%.

Suppose the ESWL machine can generate shockwaves when it detects the initial point of the respiratory regularity and the maximal inhalation time point. In that case, the stone can be positioned at the same lowest level, and the hit rates will be increased. The sensor to identify the respiratory regularity can create a signal to generate shockwaves, and the hit rates can be maximized without checking the stone location with ultrasonography and fluoroscopy.

In the occurrence of an event that changes the respiratory regularity, the shockwave generation may stop, and it can restart when detecting respiratory regularity again. For example, we observed five types of respiratory events during ESWL procedures: (1) cough, (2) sleep, (3) mask off, (4) hyperventilation or tension, and (5) conversation or body movement. In the case of regular breathing, the breathing waveform was consistent and had regular respiratory durations. On the other hand, when an event occurred, the waveform varied irregularly according to the event. Then, the stone inside the kidney moved irregularly, and the shockwave generation was unnecessary. If respiratory regularity changes, the shockwave generation may stop, and it can restart when detecting respiratory regularity again.

### ESWL outcomes according to other settings

According to previous investigations, the current standard has scaled down from 120 to 60 shockwaves per minute [2]. However, many centers have recently adopted the dual-frequency procedure of 60 and 90 Hz to shorten the treatment time [16, 17]. . In our in-vitro experiments, neither fragmentation efficiency nor cavitation at 1 Hz was superior to those at 1.5 or 2 Hz in the case of fast or intermediate respiration. Our slow respiration simulation shows slow breathing likely yields better results, as evident in higher stone fragmentation. Although the hit rates did not differ significantly across the settings when not adjusted for respiration in this study, there might be a lingering effect with slow respiration due to a slower motion of the stone, which allows more cavitation bubbles to surround the stone.

A previous investigation also showed that respiratory motion caused a reduced hit rate in an in-vitro study [18]. Another group speculated that pronounced respiratory movements provided a more extended period of shockwave focusing on the stone, and they, therefore, recommended using an abdominal belt as a means of movement reduction. Thus, the relationship between slow respiration and better stone fragmentation merits additional investigation, and we should attempt to use respiration to our advantage. However, no evidence applying an abdominal belt increases respiratory regularity.

The shockwave frequency that affects the hit rates on the stones may include frequency. An interesting factor for the reduced hit rate is the misalignment of a stone at the initiation of shockwave generation. During our experiments with fast respiration at 1 Hz, we observed that the hit rates of the shockwaves generated at the beginning of respiration were higher than that of the shockwaves that were not developed at the start of respiration (no data shown). This may suggest that the agreement between the stone location and the beginning of the shockwave generation is essential for increasing the hit rates.

### Safety of ESWL

During ESWL, physicians have attempted to reduce such pain [9, 19] by administrating analgesics [20] and diverting patients’ attention to audiovisual content [21] or music [19, 22, 23]. The phantom gel damage showed no significant differences in the slow, intermediate, and fast respiration groups without respiration adjustment. However, the slow respiration group showed less gel damage than the intermediate and fast groups, and the regular group showed less pain than the irregular group. The results suggest that shockwave generation according to respiratory regularity may contribute to reducing pain among patients undergoing ESWL. If the intensity is high and the ESWL time is extended, the number of delivered shockwaves increases, and it is reasonable that the stone-free rate increases regardless of complications [24].

### Limitations

First, comparing the stone fragmentation efficiency according to each respiratory duration and shockwave settings was impossible, as the respiratory durations of all patients varied from 2.9 to 5.5 s. Moreover, further study with a larger number of patients would be helpful to show the comparative study between the stone fragmentation efficiency and the other shockwave generation settings.

## Conclusions

Stone fragmentation efficiency was higher in the respiration-adjusted model than in the non-adjusted model. When adjusted for respiration and applying the shockwave frequency to 1 Hz, the stone fragmentation efficiency increased according to the increase in respiration cycles. The low frequencies showed higher hit rates than 2 Hz without respiration adjustment. Moreover, the stones’ fragmentation efficiency was highest in slow respiration. Misalignment of stones at the start of the shockwave generation can also reduce the hit rate.

‘Respiratory movement’ itself can compromise the efficiency of ESWL, and many urologists already recognize this. However, this study tried to analyze how much proportion of respiratory regularity could be seen and how we could define “Respiratory regularity (%)” for the first time in the world. Therefore, in the clinical setting, a high portion of patients exhibited regular breathing during ESWL. The stone fragmentation of the regular respiration group was higher than that of the irregular group. Our study reveals the potential for a new ESWL machine with a triggered shockwaves generator, a stone location detector, and a respiratory regularity sensor.

## Data Availability

Raw data are available upon request to the corresponding author.

## Abbreviations

ESWL: Extracorporeal Shock Wave Lithotripsy
RR: Respiratory Rates
